# Glycated hemoglobin A1C and vitamin D and their association with diabetic retinopathy severity

**DOI:** 10.1038/nutd.2017.30

**Published:** 2017-06-12

**Authors:** M Long, C Wang, D Liu

**Affiliations:** 1 Department of Medicine Endocrinology & Metabolism, The Second Affiliated Hospital of Chongqing Medical University, Chongqing, China

## Abstract

**Objectives:**

This retrospective, population-based, cross-sectional study evaluated the association between vitamin D deficiency and retinopathy severity in diabetic patients with poorly or well controlled glycaemia. Other potential risk factors for diabetic retinopathy severity were also assessed.

**Methods:**

The National Health and Nutrition Examination Survey (NHANES) 2005–2008 data were used for the study. Outcomes assessed included retinopathy severity, HbA1c levels, socioeconomic, behavioral, and biological factors. Univariate and multivariate regression analysis was used to evaluate association of different parameters with retinopathy severity. The interaction among HbA1c control, vitamin D deficiency, and retinopathy severity were also explored.

**Results:**

The population included 842 adults (52.8% women) with mean age of 61.2 years. Retinopathy was detected in 301 subjects (35.7%). Mild non-proliferative retinopathy (NPR) was present in 195 subjects (23.2%), severe non-proliferative and proliferative retinopathy in 106 subjects (12.6%). Multivariate ordinal regression analysis found being male (odds ratio (OR): 1.602, *P*=0.001), increased duration of diabetes (OR: 1.072, *P*=3.77E−7) and poorly controlled HbA1c (OR: 3.522, *P*=2.00E−5) were associated with greater retinopathy severity. The association between vitamin D deficiency and retinopathy severity only found in diabetic patients with well controlled glycaemia.

**Conclusions:**

The findings of this study indicate that vitamin D deficiency associated with severe diabetic retinopathy in patients with well controlled diabetes. The findings provide possible relationship for the previous conflict results, and highlight the need for controlling modifiable risk factors to reduce the development of sever diabetic retinopathy.

## Introduction

Diabetic retinopathy is the most common complication of diabetes and is the leading cause of blindness in persons aged 20 to 65 years.^1, 2^ Data from 2005 to 2008 National Health and Nutrition Examination Survey (NHANES 2005–2008) found that the risk of diabetic retinopathy affects 28 to 30% of people with diabetes,^3^ which is about 3.8% of the United States population.^4^ The prevalence of diabetic retinopathy was observed to be 37% in newly detected diabetes patients and 18% when considering all diabetic cases among those aged 40 years and older.^5^

Diabetic retinopathies that can result in vision loss include severe non-proliferative retinopathy (NPR), macular edema and retinal neovascularization. Several studies have investigated risk factors associated with diabetic retinopathy and identified both modifiable and non-modifiable risk factors for the presence and severity of diabetic retinopathy.^4, 6, 7, 8, 9, 10^ Modifiable risk factors that have been identified include the levels of blood glucose, blood pressure, serum lipids, obesity, alcohol and smoking.^7, 8^ Other modifiable risk factors include level of education and use of vitamin D or calcium supplements^7, 8^ Non-modifiable risk factors are duration of diabetes, gender and age.^7, 8^ Other independent variables include type of diabetes and family history of diabetic retinopathy.^9^

Vitamin D deficiency is common worldwide and studies indicate the overall prevalence rate of vitamin D deficiency in US adults was 41.6%.^11^ Vitamin D deficiency has been implicated in the pathogenesis and progression of diabetes and may have a role in development and severity of diabetic retinopathy.^12, 13, 14, 15, 16, 17^ However, study results have been inconsistent with respect to the association of vitamin D and diabetic retinopathy,^1, 18, 19, 20, 21, 22, 23, 24, 25, 26, 27^ and the reasons for differences in study finding are unclear.

Identifying risk factors that influence the severity of diabetic retinopathy is necessary for development of medical strategies to lessen disease progression and prevent visual loss. The importance of this is supported by the observation that between 1985 and 2009, a reduction in the rates of proliferative diabetic retinopathy and severe visual loss was observed,^28^ which was attributed to a greater awareness and management of risk factors of diabetic retinopathy.^28^ To further characterize risk factors that influence the presence and progression of diabetic retinopathy, we performed a retrospective study that used NHANES 2005–2008 database to investigate whether the level of glycemic control influenced what risk factors affect severity of diabetic retinopathy.

## Materials and methods

### Data source

This was a cross-sectional study that assessed the relationship between different factors and the severity of diabetic retinopathy stratified by glycemic control using individual patient-level data from the NHANES 2005–2008 (Centers for Disease Control and Prevention (CDC), National Center for Health Statistics (NCHS). Hyattsville, MD: U.S. Department of Health and Human Services). The NHANES program began in the early 1960s, and has been conducted as a series of surveys focusing on different population groups and health topics. The sample for the NHANES survey is selected to represent the United States population of all ages. Further information about background, design and operation are available on the NHANES website.^29^ The survey and data collection was approved by the NHANES Institutional Review Board (IRB), and the NCHS Research Ethics Review Board (ERB; Protocol#98-12, Protocol#2005-06, and Protocol #2011–17) https://www.cdc.gov/nchs/nhanes/irba98.htm. All of the NHANES data are de-identified and meet the circumstances described in Policy #39, Research Using Publicly Available Datasets (Secondary Analysis) for use without application to IRB.

### Study population

The study population included diabetic patients. Diabetes was defined as a self-report or having been told by a doctor or health professional that they had diabetes or sugar diabetes. Patients who responded ‘yes’ were classified as having a diagnoses of diabetes. Eligible diabetic patients were aged ⩾40 years during the period of 2005–2008. Pregnant women, individuals without completed retinopathy grading, or those with invalid HbA1c values were excluded from the analysis.

### Study variables

#### Retinopathy severity

Diabetic retinopathy was defined as the presence of one or more retinal micro aneurysms or retinal blot hemorrhages with or without more severe lesions using the ophthalmic digital imaging system (retinal photography). Retinopathy severity was determined by assessment of the retinopathy level based on NHANES Grading Protocol of the more affected eye.^30^ Retinopathy severity was further categorized as: no retinopathy, mild non-proliferative retinopathy (NPR) and severe NPR/proliferative, which was indicated as ‘OPDURL4’ in the NHANES database. Detailed descriptions of the protocol are provided in the NHANES Digital Grading Protocol, Retinopathy Sub-section.^30^

#### Glycemic control

HbA1c levels, which can reflect a person’s average level of blood glucose over the past three months, were evaluated by boronate affinity high-performance liquid chromatography (HPLC) as described in the NHANES Laboratory Procedure Manual, Glycohemoglobin.^31^ We stratified glycemic control into well controlled (HbA1c <7%) and poorly controlled (HbA1c⩾7%).

#### Socioeconomic

Age, gender, race/ethnicity, education (less than 9th grade education, Grades 9 to 12, and college and above) and poverty income ratio (a ratio of family income to poverty threshold) were obtained from the NHANES database.

#### Behavioral

Smoking was classified as never, former or current smoker. Subjects who never had at least 100 cigarettes in their life were defined as non-smoker. Those who had at least 100 cigarettes but not smoke now were former smoker. Those who response ‘yes’ in the question ‘Do you smoke now?’, were defined as current smoker. The use of vitamin D or calcium supplement usage was also queried from dietary supplement questionnaire. Intake estimations were calculated based on the information of vitamin D or calcium content of supplements and the frequency, types and amounts of supplements consumed.

#### Biological measurements

Duration of diabetes was calculated from the reported age at screening minus the age of the subject when first told he/she had diabetes. Hypertension was defined by response to the following question: ‘Were you told on two or more different visits that you had hypertension, also called high blood pressure?’ Family history of diabetes was determined by the answer to the following question: ‘Including living and deceased, were any of your biological relatives, that is, blood relatives, including grandparents, parents, brothers, and sisters, ever told by a health professional that they had diabetes?’

Serum 25-hydroxy vitamin D (nmol l^−1^) were measured at the National Center for Environmental Health, CDC, Atlanta, GA using the DiaSorin RIA kit (Stillwater, MN, USA) in NHANES 2005-2006. However, in the NHANES 2007–2010, liquid chromatography-tandem mass spectrometry (LC-MS/MS) was used. We used the LC-MS/MS-equivalent data for the present study.^32^ Due to the fact there is still some controversy on the best classification of Vitamin D status,^33, 34^ we used the clinically relevant definition of serum 25(OH)D levels (<50 nmol l^−1^) to define vitamin D deficiency in the present study.

Serum total cholesterol, serum triglyceride, and serum HDL were measured enzymatically using a series of coupled reactions performed via the Hitachi 704 Analyzer, which was serviced by Roche Diagnostics. LDL-cholesterol was calculated from measured values of total cholesterol, triglycerides, and HDL cholesterol using the equation: [LDL-chol]=[total chol]−[HDL-chol] - [TG]/5. Detailed descriptions of blood collection and processing procedures were provided in the NHANES Laboratory/Medical Technologists Procedures Manual or NHANES website.^29, 35^ Overweight was dichotomized, and defined by body mass index (BMI) (⩾25 kg m^−2^).

#### Statistical analysis

All analyses included full sample two-year Mobile Exam Center (MEC) exam weight (WTMEC2YR), stratum, and primary sampling units (PSU) per recommendations from NCHS, to address oversampling, non-response, non-coverage and to provide nationally representative estimates.

Differences in categorical variables by levels of retinopathy were determined using *χ*^2^-test of independence, and differences among groups in continuous variables were examined using the Complex Samples General Linear Model (CSGLM). Data of demographic and basic characteristics were expressed as mean (s.e.) for continuous variables or unweighted counts (weighted %) for categorical variables. Univariate and multivariate ordinal regression analysis was performed to determine the significant factors associated with retinopathy severity. Variables having a *P*-value<0.05 in the univariate analysis were selected and evaluated by multivariate ordinal regression models. The multiple comparison adjustment was used the Bonferroni correction. In addition, the interaction of HaA1c with vitamin D status was also assessed. All statistic assessments were two sided and evaluated at the 0.05 level of significance. Statistical analyses were performed using the statistical software package SPSS complex sample module version 22.0 (IBM Corp, Armonk, NY, USA).

## Results

### Study population characteristics

The study sample included 842 adults. Using NHANES 2-year MEC exam sample weight, the estimated population size was 12 214 485 participants. The study population contained of 424 women (52.8%) and the mean (±s.e.) age of the study participants was 61.24±0.46 years. Retinopathy was detected in 301 subjects (35.7%). Mild NPR was present in 195 subjects (23.2%), severe NPR and proliferative diabetic retinopathy in 106 subjects (12.6%).

Table 1 shows the demographic, socioeconomic, comorbidity and baseline characteristics by level of retinopathy. Significant differences in gender, race, duration of diabetes and level of HbA1c was observed among the different levels of retinopathy severity (all *P*-values<0.05). A higher percentage of females were present in the group of subjects with no retinopathy (*P*=0.006). In all retinopathy groups most of the population were non-Hispanic Whites (range, 54.3 to 66.2% *P*=0.004). Subjects with severe NPR/proliferative retinopathy had the longest duration of diabetes (18.2 years, *P*=1.03E−8) followed by those with mild NPR (14.4 years) then subjects with no retinopathy (7.5 years). A higher percentage of poorly controlled HbA1C in the severe NPR/proliferative retinopathy group (71.6% compared with 65.3% for mild NPR and 33.1% for no retinopathy, *P*=6.33E−8).

### Factors associated with retinopathy severity

Regression analysis was performed to identify factors in the entire study population that were associated with retinopathy severity. The result of univariate ordinal regression analysis indicates gender (male), ethnicity (black), duration of diabetes and HbA1c poor controlled were positively associated with retinopathy severity (all *P*<0.05, Table 2). In multivariate ordinal regression analysis, male gender was associated with increased severity of retinopathy (odds ratio (OR): 1.602, *P*=0.001), increased duration of diabetes (OR: 1.072, *P*<0.001), and poorly controlled HbA1c levels (OR: 3.522, *P*<0.001) were positively associated with increased severity of retinopathy.

### HbA1c and vitamin D deficiency and their interaction with diabetic retinopathy severity

**Figure 1:**
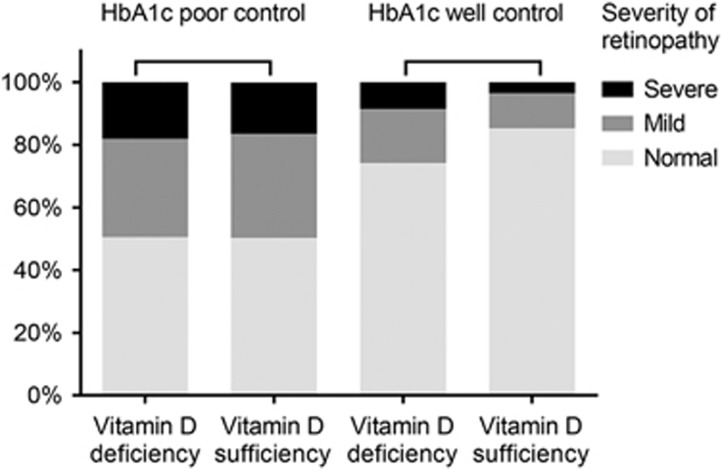
The stacked bar chart of the severity of retinopathy in patients with poorly controlled or well controlled glycaemia with and without vitamin D deficiency (Note: The percentages were adjusted by the 4-year sample weights from NHANES 2005−2008).

Figure 1displayed a stacked par chart that examined the severity of retinopathy in patients with poorly controlled or well controlled glycaemia with and without vitamin D deficiency. The distribution of retinopathy severity were similar between vitamin D deficiency and sufficiency in patients with poorly controlled glycaemia. However, the distribution of retinopathy severity in patients with well controlled glycaemia is another story. Among those well controlled patients, the percentage of severe or mild retinopathy were higher in vitamin D deficiency group than in vitamin D sufficiency group, which were 8.5% vs 5.3%, and 17.3% vs 13.4%, respectively. In addition, there was a significant interaction of HbA1C with vitamin D deficiency (*P*=0.038). In addition, after adjusting gender, ethnicity and duration of diabetes, the interaction of HbA1 with vitamin D deficiency significantly affected retinopathy severity (*P*=0.029) (Table 3).

## Discussion

The aim of this study was to investigate risk factors associated with diabetic retinopathy severity, specifically the study evaluated the relationship between vitamin D status and severity of diabetic retinopathy in patients with good or poor glycemic control. We found that being male, increased duration of diabetes and increased HbA1c levels were positively associated with increased severity of diabetic retinopathy.

The idea to stratify the patients into two groups based on glycemic control was based on the fact that HbA1c levels impact the presence of diabetic retinopathy.^36^ The accepted international standard for the diagnosis cutoff point has been determined to be >6.5%^(ref. 36)^ and >7% for DM patients.^37^ HbA1c levels can impact a number of molecular and cellular processes that may influence retinopathy development and severity, including microvasculature complication, ischemia, increased levels of vascular endothelial growth factor (VEGF) and inflammation.^6, 36^

Our study was consistent with some, but not all, prior studies with respect to other risk factors for diabetic retinopathy severity (for example, being male, HbA1c levels and duration of diabetes), inconsistencies are also observed. One pooled analysis of population-based studies included 35 studies with 22 896 subjects.^10^ In the study, the overall presence for any severity of diabetic retinopathy was 34.6%. The prevalence of any severity of diabetic retinopathy increased with duration of diabetes, blood pressure and was lower in patients with type 2 compared with type 1 diabetes.^10^ A cross-sectional study found a significant association between diabetic retinopathy and serum triglyceride and cholesterol levels, and duration of diabetes.^9^ In contrast, we did not find an association of triglycerides and cholesterol levels with diabetic retinopathy severity in this study. Another study assessed factors associated with the prevalence and severity of diabetic retinopathy in patients with type 2 diabetes.^6^ They found that poor glycemic control (>8%), microalbuminuria, hypertension, BMI >35 kg m^−2^, and male gender were significantly associated with retinopathy. Similar to our findings, they found that HbA1c level was a strong predictor of severe retinopathy. Other strong predictors of retinopathy severity were the presence of micro- and macroalbuminuria.

Vitamin D deficiency has been implicated in the progression and pathogenesis of diabetes.^12, 13, 14, 15, 38^ Vitamin D levels are also correlated with metabolic syndrome, obesity, insulin resistance, and cardiovascular disease.^12, 13, 14, 15, 38^ Vitamin D deficiency potentially may impact the development of diabetic retinopathy in several ways, such as affecting insulin secretion, insulin sensitivity, influencing inflammation, immunosuppression, microvascular and macrovascular events, and angiogeneisis.^16, 17, 39, 40, 41, 42, 43^

A number of recent studies have evaluated the association of vitamin D with diabetic retinopathy. Four studies found no association of vitamin D status with diabetic retinopathy,^19, 24, 26, 27^ and more than five studies found a relationship of vitamin D levels with diabetic retinopathy.^18, 20, 21, 22, 23, 25^ A cross-sectional study used data of patients with diabetes (>40 years of age) from the NHANES (2008–2012).^26^ They found that although the percentage of individuals with vitamin D deficiency increased with retinopathy severity, regression analysis did not demonstrate a significant association between the two variables (*P*=0.07).^26^ Similarly, the EURODIAB prospective complication study found that 1 nmol/L higher 25(OH)D_2_ and 10 nmol l^−1^ higher 25(OH)D_2_ serum levels were not associated with non-proliferative and proliferative retinopathy.^27^ A cross-sectional study in adults with type 1 and type 2 diabetes found no difference in the levels of serum vitamin D across retinopathy severity; no diabetic retinopathy (39%), background diabetic retinopathy (37%), preproliferative diabetic retinopathy (21%), and proliferative diabetic retinopathy (3%).^24^ Logistic regression analysis found no significant association between retinopathy severity and serum vitamin D concentration.^24^ A clinic-based cross-sectional study in patients with type 2 diabetes >20 years of age found no difference in vitamin D levels between patients without diabetic retinopathy and those with the ocular disease.

In contrast, Askoy *et al.* (2000) found a correlation between lower active vitamin D (1,25-dihyroxyvitamin D_3_) levels and increased retinopathy. Similarly, Gunger *et al.* (2015) compared 50 patients with early-stage diabetic retinopathy with vitamin D deficiency with 50 patients with early stage diabetic retinopathy without vitamin D deficiency.^21^ They found vitamin D deficiency was associated with early retinal nerve fiber layer thinning.^21^ A study that sampled 18 363 patients from NHANES (2008–2012) found vitamin D was associated with retinopathy.^22^ Similarly, a study in a Chinese population of patients with type 2 diabetes found using logistic regression analysis that vitamin D deficiency was an independent risk factor for diabetic retinopathy and vision-threatening diabetic retinopathy.^23^ A study in a Japanese population of patients with type 1 diabetes also found a relationship of vitamin D deficiency with diabetic retinopathy.^25^

Differences across the earlier studies may be due to, at least in part, from the fact that none of the earlier studies evaluated the impact of HbA1c levels on the association of vitamin D with diabetic retinopathy. Our results indicate insufficient vitamin D may increase the risk of severe diabetic retinopathy only in patients with well controlled glycaemia. Subgroup analysis that examined the severity of retinopathy in patients with poorly controlled or well controlled glycaemia with and without vitamin D deficiency found that vitamin D deficiency and dichotomous HbA1c have significant interaction and greater NPR severity was associate with vitamin D deficiency for both glycaemia populations. The fact that the risk factors in our study differed between the well-controlled and poorly-controlled glycemic subgroups strongly suggests that future studies should consider this issue within their study design.

One of the strengths of the present study is that it utilized a survey database that was representative of the population of the United States, and hence the findings are likely generalizable to the overall United States population. In addition, we explored whether glycemic control influenced the impact of vitamin D deficiency as a risk factor for diabetic retinopathy. However, there are several limitations to this study. The study was cross-sectional rather than longitudinal in design, and thus causality cannot be established. We only evaluated a subset of factors that are known to be related to diabetes. In addition, the study could not distinguish between patients with type 1 or type 2 diabetes. The number of patients with moderate, and in particular, proliferative diabetic retinopathy was small. Also, the identification of cases of diabetes, family history of diabetes, and presence of hypertension were self-reported, which may induce fatally flawed.^44, 45^ The study was also limited by the fact that the presence of diabetes, hypertension, and sedentary behavior were determined by patient self-report and that did not distinguish between Type 1 and Type 2 diabetes. The use of self-reporting for indicating the presence of diabetes is supported by several studies that self-report can be a reliable estimated for the presence of diabetes.^46, 47, 48^ In addition, although HbA1c can be used to diagnose the disease, it is not always accurate in assessing glycemia in some situations and the diagnostic threshold of 6.5% is controversial.^49, 50^ Fasting blood glucose is also used to diagnose diabetes but requires two separate blood tests in which the glucose levels are⩾126 mg/dl. We also performed a post-hoc analysis to assess using the NHANES data the accuracy of self-reported diabetes. We found that for patients with HbA1c ⩾6.5%, 63% (530/750) correctly reported having diabetes and in patients with FBG ⩾126 mg dl^−1^ 66% (285/436) correctly indicated they had diabetes.

In conclusion, this study identified that vitamin D deficiency is associated with severity of retinopathy only in diabetic patients with good glycemic control. These findings may give important insight into conflicting findings from prior studies with respect to the role of vitamin D in diabetic retinopathy in which populations were not stratified by HbA1c levels. Our findings, and those of others, highlight the need for medical treatment and management of diabetic retinopathy to focus on certain modifiable variables, such as blood sugar control, and vitamin D supplemental, so as to reduce the risk of developing severe ocular disease. However, this was a cross-sectional, retrospective associative study based on self-report, additional studies are necessary to further evaluate the impact of glycemic control on risk factors that influence the presence and severity of diabetic retinopathy.

## Figures and Tables

**Table 1:** Demographic and basic characteristics from NHANES 2005–2008 (Unweighted *n*=842, Weighted *N*=12 214 485)^a^

*Characteristics*	*No retinopathy*	*Mild NPR*	*Severe NPR/proliferative retinopathy*	P-*value*
Unweighted, *n*	541	195	106	
Age (years)	60.63±5.50	63.25±0.99	60.86±1.24	0.108
Gender, *n* (%)				0.006^b^
Male	253 (43.2%)	114 (56.9%)	51 (51.8%)	
Female	288 (56.8%)	81 (43.1%)	55 (48.2%)	
				
*Ethnicity,* n *(%)*				0.004^b^
Non-Hispanic White	231 (66.2%)	82 (67.3%)	26 (54.3%)	
Non-Hispanic Black	141 (14.3%)	57 (16.9%)	48 (30.6%)	
Mexican American	105 (7.5%)	36 (7.3%)	22 (10%)	
Other Hispanic	47 (5.1%)	16 (7.1%)	8 (2.4%)	
Other	17 (6.8%)	4 (1.5%)	2 (2.8%)	
				
*Education,* n *(%)*				0.953
Less than 9th grade	106 (13.3%)	50 (15.7%)	22 (14.0%)	
Grades 9–12	244 (43.3%)	81 (42.5%)	49 (43.7%)	
College or above	191 (43.4%)	64 (41.9%)	35 (42.3%)	
Poverty income ratio	2.86±0.09	2.67±0.15	2.87±0.22	0.491
Duration of diabetes (years)	7.53±0.41	14.41±0.83	18.20±1.33	1.028E−8^b^
				
*Smoking,* n *(%)*				0.090
Current smoker	83 (14.7%)	39 (20.7%)	11 (9.6%)	
Former smoker	211 (38.8%)	70 (33.9%)	37 (29.6%)	
Non-smoking	246 (46.4%)	86 (45.4%)	58 (60.8%)	
				
*Overweight,* n *(%)*				0.942
Yes	467 (88.2%)	196 (89.0%)	90 (88.0%)	
No	66 (11.8%)	26 (11.0%)	15 (12.0%)	
				
*Hypertension,* n *(%)*				0.712
Yes	327 (89.2%)	115 (88.0%)	69 (83.8%)	
No	44 (10.8%)	19 (12.0%)	9 (16.2%)	
				
*Vitamin D deficiency,* n *(%)*				0.286
Yes	228 (36.6%)	85 (43.5%)	63 (49.0%)	
No	279 (63.4%)	92 (56.5%)	39 (51.0%)	
				
*HbA1C poor control,* n *(%)*				3.202 E−8^b^
Yes	119 (33.1%)	122 (65.3%)	81 (71.6%)	
No	342 (66.9%)	73 (34.7%)	25 (28.4%)	
HbA1C (%)	6.78±0.07	7.64±0.15	8.21±0.24	6.328 E−7^b^
Triglyceride (mmol l^−1^)	2.25±0.25	1.72±0.10	2.22±0.32	0.114
Total cholesterol (mmol l^−1^)	4.82±0.08	4.61±0.11	4.79±0.20	0.226
HDL (mmol l^−1^)	1.25±0.02	1.28±0.02	1.25±0.06	0.586
LDL (mmol l^−1^)	2.53±0.05	2.55±0.12	2.64±0.21	0.833
Vitamin D supplement (mcg)	12.08±0.60	14.47±2.61	13.78±3.39	0.682
Calcium supplement (mg)	410.89±39.94	371.33±51.49	477.54±119.91	0.730

Abbreviations: NHANES, National Health and Nutrition Examination Survey; NPR, non-proliferative retinopathy.

Values are mean±s.e. for continuous variables or unweighted counts (weighted %) for categorical variables. A *P*-value displays in scientific notation if *P*<0.0005.

a Data are weighted according to the National Health and Nutrition Examination Survey protocol.

b Significant difference among the 3 levels of retinopathy, *P*<0.05.

**Table 2:** Ordinal regression analysis of factors associated with retinopathy severity from NHANES 2005-2008 (Unweighted *n*=842, Weighted *N*=12 214 485)

	*Univariate*		*Multivariate*	
*Characteristics*	*Odds ratio (95% CI)*	P-*values*	*Odds ratio (95% CI)*	P-*values*
Age	1.103 (0.997, 1.029)	0.109		
Gender (Male vs female)	1.567 (1.182, 2.078)	0.003*	1.602 (1.220, 2.103)	0.001*
*Ethnicity*		0.047*		0.178
Black vs White 230)	1.698 (1.020, 2.824)	0.042	1.177 (1.012, 3.120)	0.046
Mexican American vs White	1.186 (0.728, 1.932)	0.481	1.152 (0.665, 1.996)	0.604
Other Hispanic vs White	1.066 (0.582, 1.951)	0.831	1.156 (0.619, 2.157)	0.639
Other vs White	0.301 (0.082, 1.104)	0.069	0.572 (0.160, 2.046)	0.378
				
*Education*		0.796		
<9th grade vs College or above	1.155 (0.677, 1.972)	0.586		
Grades 9–12 vs College or above	1.022 (0.633, 1.650)	0.926		
				
Poverty income ratio	0.959 (0.858, 1.072)	0.450		
Duration of diabetes	1.077 (1.049, 1.106)	**2.452** E−6*	1.072 (1.049, 1.096)	**3.770** E−7*
				
*Smoking*		0.379		
Current smoker vs non-smoker	1.031 (0.697, 1.527)	0.873		
Former smoker vs non-smoker	0.771 (0.433, 1.372)	0.771		
				
Overweight (Yes vs No)	1.042 (0.646, 1.681)	0.862		
Hypertension (Yes vs No)	0.756 (0.370, 1.543)	0.429		
Vitamin D deficiency (Yes vs No)	1.450 (0.867, 2.427)	0.150		
HbA1C poor control (Yes vs No)	4.081 (2.646, 6.294)	**2.129** E−7*	3.522 (2.113, 5.872)	**2.000** E−5*
Triglyceride	0.918 (0.793, 1.063)	0.242		
Total cholesterol	0.917 (0.785, 1.071)	0.263		
HDL	1.157 (0.681, 1.967)	0.579		
LDL	1.068 (0.844, 1.350)	0.573		
Vitamin D supplement (mcg)	1.013 (0.992, 1.035)	0.216		
Calcium supplement (mg)	1.000 (0.999, 1.001)	0.979		

Abbreviations: CI, confidence interval; NHANES, National Health and Nutrition Examination Survey.

A *P*-value displays in scientific notation if *P*<0.0005.

*Significant difference in the ordinal regression models, *P*<0.05.

**Table 3:** Effects of HbA1c poor control and vitamin D, and their interaction in the ordinal regression from NHANES 2005-2008 (Unweighted *n*=842, Weighted *N*=12 214 485)

	*Crude model*		*Adjusted model*	
	*Odds ratio (95% CI)*	P-*values*	*Odds ratio (95% CI)*	P-*values*
HbA1c poor control	5.608 (3.323, 9.464)	**1.614** E−7*	5.102 (2.799, 9.300)	**4.618** E−6*
Vitamin D deficiency	2.061 (1.265, 3.357)	0.005*	2.226 (1.359, 3.648)	0.002*
HbA1c poor control × Vitamin D deficiency^a^	0.496 (0.256, 0.959)	0.038*	0.442 (0.213, 0.914)	0.029*
Gender (Male vs Female)			1.546 (1.140, 2.097)	0.007*
				
*Ethnicity*				0.331
Black vs White			1.587 (0.966, 2.606)	0.067
Mexican American vs White			1.114 (0.643, 1.927)	0.692
Other Hispanic vs White			1.133 (0.596, 2.153)	0.694
Other vs White			0.454 (0.094, 2.199)	0.315
				
Duration of diabetes (years)			1.070 (1.045, 1.095)	**1.466** E−6*

Abbreviations: CI, confidence interval; NHANES, National Health and Nutrition Examination Survey.

*Significant difference in the ordinal regression models, *P*<0.05.

A *P*-value displays in scientific notation if *P*<0.0005.

a Interaction between HbA1c poor control and Vitamin D deficiency.
